# A Taxonomic Study of *Candolleomyces* Specimens from China Revealed Seven New Species

**DOI:** 10.3390/jof10070499

**Published:** 2024-07-19

**Authors:** Xi-Xi Han, Dorji Phurbu, Guo-Fei Ma, You-Zhi Li, Yu-Jiao Mei, Dong-Mei Liu, Fu-Cheng Lin, Rui-Lin Zhao, Naritsada Thongklang, Bin Cao

**Affiliations:** 1 School of Science, Mae Fah Luang University, Chiang Rai 57100, Thailand; 2 Center of Excellence in Fungal Research, Mae Fah Luang University, Chiang Rai 57100, Thailand; 3 State Key Laboratory of Mycology, Institute of Microbiology, Chinese Academy of Sciences, Beijing 100101, China; 4 Tibet Plateau Institute of Biology, Lhasa 850000, China; 5 Shennongjia National Park Administration, Shennongjia 442421, China; 6 Hubei Provincial Key Laboratory on Conservation Biology of the Shennongjia Golden Snub-Nosed Monkey, Shennongjia 442421, China; 7 Institue of Ecology, Chinese Research Academy of Environmental Sciences, Beijing 100012, China; 8 State Key Laboratory for Managing Biotic and Chemical Treats to the Quality and Safety of Agro-Products, Institute of Plant Protection and Microbiology, Zhejiang Academy of Agricultural Sciences, Hangzhou 310021, China; 9 Institute of Digital Agriculture, Zhejiang Academy of Agricultural Sciences, Hangzhou 310021, China; 10 College of Life Sciences, University of Chinese Academy of Sciences, Beijing 100049, China

**Keywords:** Psathyrellaceae, multigene, phylogeny, taxonomy

## Abstract

Based on phylogenetic analysis, *Candolleomyces* (Psathyrellaceae, Agaricales) was established with *Psathyrella candolleana* as the type species. The basidiomes range from small to large and are typically terrestrial, lignicolous, and rarely fimicolous. We analysed the *Candolleomyces* species collected during five years in China, and based on morphological and molecular data (nrITS, nrLSU, and *tef-1α*), we propose seven new *Candolleomyces* species viz. *C. brevisporus*, *C. gyirongicus*, *C. lignicola*, *C. luridus*, *C. shennongdingicus*, *C. shennongjianus*, and *C. sichuanicus*. Full descriptions, colour photographs, illustrations, phylogenetic analyses results, and comparisons with related *Candolleomyces* species of the new taxa are provided. This study enriches the species diversity of *Candolleomyces* in China.

## 1. Introduction

The recently established genus *Candolleomyces* D. Wächt. & A. Melzer is characterised by small to large basidiomes, being terrestrial, lignicolous, and rarely fimicolous, the veils are often fibrillose, scaly, or granulose, but very fugacious, stipes occasionally with an annulus, basidiospores are medium-sized, it is pale to medium-dark in colour, with a central, usually invisible, germ pore, the presence of cheilocystidia, and absence of pleurocystidia. Most of the species of *Candolleomyces* historically belonged to *Psathyrella* (Fr.) Quél. [1,2]. Smith (1972) and Kits van Waveren (1985) made remarkable works on *Psathyrella* from Europe and North America, and clarified the morphological species concept within this genus [3,4]. The former identified 11 subgenera viz. *Candolleana*, *Conocybella*, *Cystopsathyra*, *Homophron*, *Lacrymaria*, *Mycophila*, *Panaeolina*, *Pannucia*, *Psathyrella*, *Psathyroides*, and *Pseudostropharia* [3,4]. Meanwhile, the latter put *Psathyrella* into two subgenera, *Psathyra* and *Psathyrella* [3,4]. Molecular phylogeny based on the nuclear ribosomal internal transcribed spacer region (nrITS) and the nuclear ribosomal large subunit ribosomal RNA gene (nrLSU) did not distinguish the species well within *Psathyrella* [5]. Therefore, two protein-coding genes viz. the translation elongator factor alpha (*tef-1α*) and beta-tubulin (*β-tub*) were also employed for species classification of *Psathyrella* [6,7,8]. Based on numerous specimen studies, and morphological and phylogenetic analyses, Wächter and Melzer [9] revised the Psathyrellaceae and divided *Psathyrella* into 18 subclades, with one of them established as the new genus *Candolleomyces* and *C. candolleanus* (Fr.) D. Wächt. & A. Melzer as the species type.

In the field, the members of *c* are similar to those species of *Psathyrella*, as they share many similar macro-morphological features and ecological niches. However, *Candolleomyces* species can be distinguished micro-morphologically by the absence of pleurocystidia, and slightly thicker-walled sphaerocysts can be observed in the veil [9]. Therefore, accurate identification requires proper examination of micromorphological characterisation and molecular phylogenetic analyses. Up to now, 43 species of *Candolleomyces* were reported worldwide [9,10,11,12,13,14,15,16,17,18,19,20]. Of these, 15 species were reported in China [11,12,19,21].

Most species of this genus are terrestrial, lignicolous, and rarely fimicolous, and can grow on rotten wood, plant debris, or litter [9,14]. Recently two species (*C. brunneovagabundus* and *C. albovagabundus*) were described to be in marine habitats [19]. *Candolleomyces* species, recognised as specialised saprophytes [9,14], were reported to be in temperate, tropical, and subtropical regions across Africa, Asia, Europe, North America, and South America [11,12,13,14,17,22]. In addition, some species of *Candolleomyces* were reported to have edible and medicinal values, while a few species were found to be poisonous fungi [23,24,25]. The type species *C. candolleanus* was reported to have medicinal value, although it may cause gastroenteritis and neurotoxicity [23,24,25]. *Candolleomyces tuberculatus* was reported to have edible value [23,24,25]. Based on poisoning incidents, *C. yanshanensis*, which was previously uncertain of its edibility, was found to be poisonous and can cause psycho-neurological disorders [26].

In the present study, we collected 31 specimens from Beijing City, Guizhou, Hubei, Sichuan, and Yunnan provinces, and the Xizang Autonomous Region in China, and conducted comprehensive phylogenetic analyses using the nrITS, nrLSU, and *tef-1α* gene regions. Based on the phylogenetic and morphological analyses, seven new species from China are proposed.

## 2. Materials and Methods

### 2.1. Morphological Characteristic Examination

Fresh specimens were collected and photographed (Canon EOS 80D, Tokyo, Japan) in the field from Beijing City, Yunnan, Sichuan, Guizhou, and Hubei provinces, and the Xizang Autonomous Region in China from 2019 to 2023. To avoid mixing or crushing, specimens were packed separately in aluminium foils. Macroscopic characteristics were recorded when fresh, including features of the pileus, veil, context, lamellae, stipe, odour, and chemical reactions. The samples were completely dried with a food drier at 50 °C, sealed in plastic bags, and deposited in the Mycological Herbarium, Institute of Microbiology, Chinese Academy of Sciences (HMAS).

Microscopic characteristics, such as basidia, basidiospores, pileipellis, and cheilocystidia, were observed under an Olympus CX31 light microscope (Olympus, Tokyo, Japan), and at least 30 measurements were made for each character. The description of morphological characteristics followed the protocols of Largent [27]. Additionally, 5% KOH and sterilised water were used for microscopic characterisation. Measured values are given as (a)b–c(d), in which a is the lowest value, b–c includes at least 90% of the values, and d is the highest value. The Q value is the ratio of the length and width of a spore [11]. The colour designation refers to the Methuen Handbook of Colour.

### 2.2. DNA Extraction, PCR and Sequencing

DNA was extracted from dried specimens using a Broad-spectrum Plant Rapid Genomic DNA Kit (Biomed, Shiyan, China). Primers ITS1 and ITS4 were used for the nuclear internal transcribed spacer (nrITS) of the rDNA region [28], LR7/LR0R were used to amplify the large subunit nuclear ribosomal DNA (nrLSU) region [29], and EF983F/EF2218R were used to amplify the translation elongation factor subunit 1 alpha (*tef-1α*) region [30]. PCR was performed in 25 µL reactions consisting of 2 µL genomic DNA, 1 µL of each forward and reverse primers, 9 µL ddH_2_O, and 12 µL 2 × Es Taq MasterMix (Beijing Cowin Biotech Co., Ltd., Beijing, China). The PCR programmes follow Zhao et al. [31] and Bau and Yan [11]. The PCR products were detected by electrophoresis and sent to BGI Genomics Co., Ltd., Shenzhen, China, for purification and sequencing.

### 2.3. Phylogenetic Analyses

Considering the results of BLAST searches against GenBank and previous studies, we analysed the nrITS, nrLSU and *tef-1α* sequences of 94 taxa. The details are presented in Table 1. The sequences were aligned by Muscle version 3.6 separately [32], then manually adjusted in BioEdit version 7.0.4 to remove the ambiguous areas [33], and assembled in PhyloSuite version 1.2.3 [34]. The final alignments were deposited in TreeBASE (study no. 31401). Maximum likelihood (ML) analysis of concatenated sequences was carried out using raxmlGUI 1.3 with a GTRGAMMA model and one thousand rapid bootstrap (BS) replicates [35]. The best partitioning scheme and evolutionary models for three pre-defined partitions were selected using PartitionFinder2 v2.1.1 [36], with greedy algorithm and AICc criterion: GTR+I+G for nrLSU, GTR+G for nrITS, and GTR+I+G for *tef-1α*. Bayesian Inference (BI) analysis was performed using MrBayes v3.2.7a [37]. Six Markov chains were run for two million generations, and trees were sampled every 100th generation. Burn-ins were determined in Tracer version 1.6 with an ESS value higher than 200, and the remaining trees were used to calculate Bayesian posterior probabilities (PP). The trees were displayed in FigTree version 1.4.0 [38].

## 3. Results

### 3.1. Phylogeny

**Figure 1:**
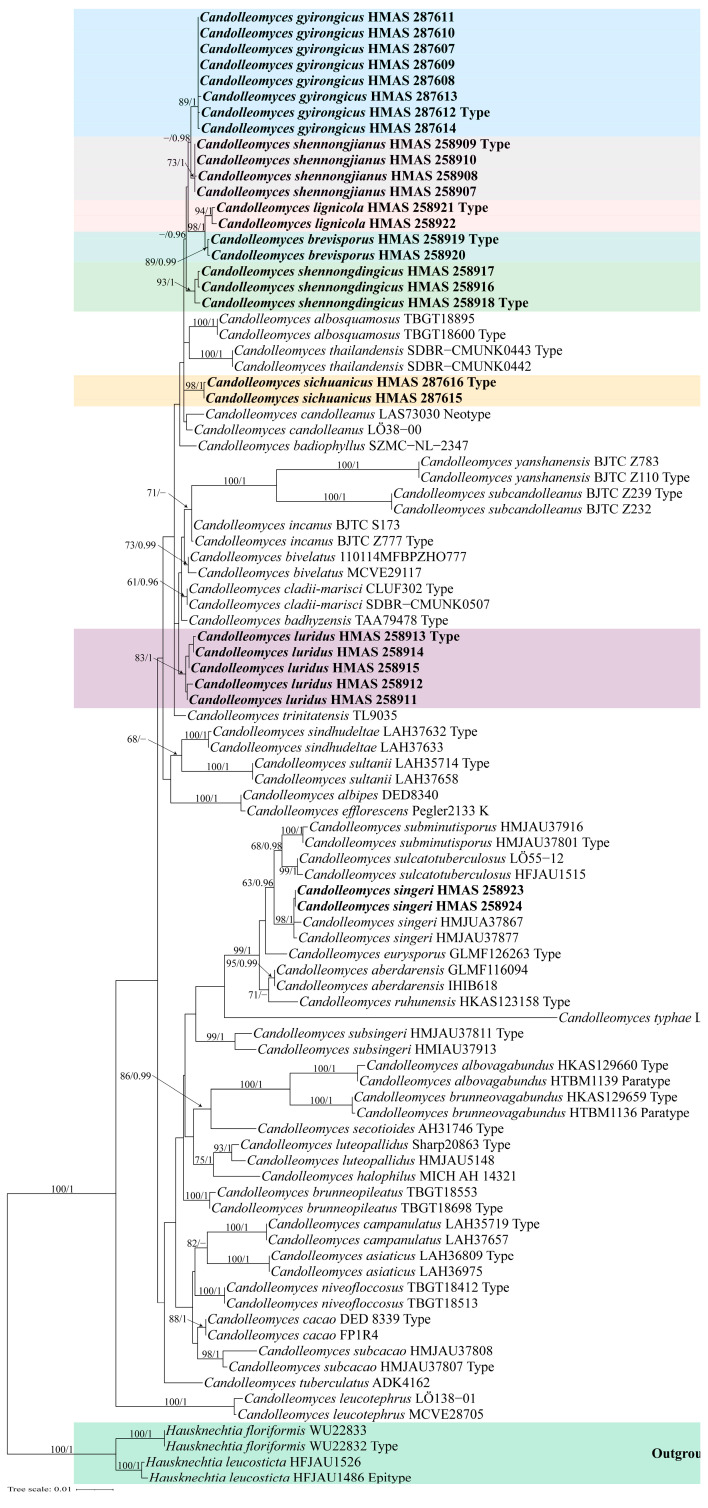
Molecular phylogenetic analyses of *Candolleomyces* species by the maximum likelihood (ML) method based on combined nrITS, nrLSU, and *tef-1α* sequences. Maximum likelihood bootstrap support values (ML) ≥ 60% and Bayesian posterior probabilities (PP) ≥ 0.95 are shown at the nodes as ML/PP. *Candolleomyces* species produced in this study are indicated in bold.

Eighty-nine specimens from 43 *Candolleomyces* species were included in the phylogenetic analyses with *Hausknechtia floriformis* (Hauskn.) D. Wächt. & A. Melzer and *H. leucosticta* (Pat.) Tkalčec, J.Q. Yan, C.F. Nie & C.K. Pradeep as outgroups. In total, 84 new sequences were generated in this study, which were from 28 specimens from China, all with the nrITS, nrLSU, and *tef-1α* sequences. The combined dataset with 3033 characters including gaps (679 for nrITS, 1322 for nrLSU, and 1032 for *tef-1α*) was included in the phylogenetic analyses. The phylogenetic tree of ML and MrBayes were almost identical. The ML tree is shown in Figure 1 with bootstrap values and Bayesian posterior probabilities indicated on the branches.

### 3.2. Taxonomy

**Figure 2:**
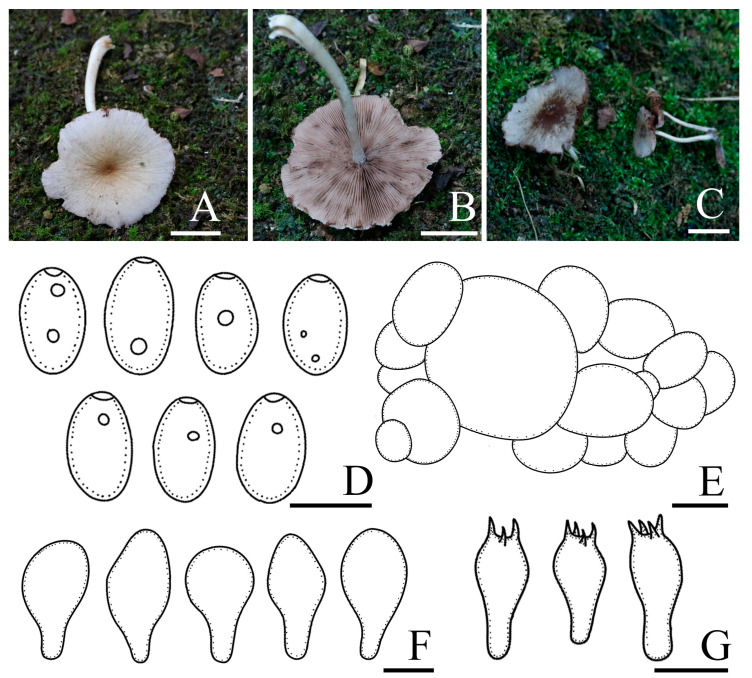
Basidiomata and microscopic features of *Candolleomyces brevisporus*. (**A**–**C**) Basidiomata: (**A**,**B**) HMAS 258919 (holotype); (**C**) HMAS 258920, (**D**) Basidiospores, (**E**) Pileipellis, (**F**) Cheilocystidia, and (**G**) Basidia. Scale bars: 10 mm (**A**–**C**); 5 μm (**D**); 20 μm (**E**); and 10 μm (**F**,**G**).

*Candolleomyces brevisporus* R.L. Zhao, B. Cao & X.X. Han, sp. nov., Figure 2.

Fungal Names: FN571747.

Holotype: CHINA. Guizhou Province, Doupeng Mountain, 26°37′41″ N, 107°36′54″ E, 1057.8 m asl, 25 September 2021, *Yang Liu* and *Chen-Hao Li*, *ZRL20211844* (holotype HMAS 258919). GenBank: OR822167 (nrITS), OR822149 (nrLSU), OR819986 (*tef-1α*).

Etymology: ‘brevisporus’ (Latin) referring to the shorter spores, a distinguishing characteristic of the species.

Diagnosis: *Candolleomyces brevisporus*, is distinguishable by its pileus, not hygrophanous. Basidiospores (5.0)5.7–6.7(7.3) × (3.3)3.7–4.2(4.5) μm, germ pores are distinct. Pileipellis is a two to three-layered irregular epithelium composed of subglobose cells. Cheilocystidia claviform to utriform, rarely pyriform.

Pileus is 12–33 mm diam, plano-convex to nearly plane, becoming slightly concave at maturity, not hygrophanous, yellowish grey (4B2) to grey (5B1), darker in the centre, occasionally with yellowish grey (5F6) veil elements, becoming white (3A1) as pileus dries. Veil is yellowish grey (5F6), dispersed, fibrillose, falling off easily. Context is thin and very fragile, the same colour as the pileus. Lamellae is moderately close, adnate to slightly adnexed, grey (5B1) to brownish orange (5C3), and edge white as basidiospores mature. Stipes are 19–37 × 1–3 mm, smooth, cylindrical, hollow, equal, and slightly yellowish white (3A2) at the apex. Odour is indistinct.

Basidiospores are (5.0)5.7–6.7(7.3) × (3.3)3.7–4.2(4.5) μm, Q = 1.4–1.7, ellipsoid to oblong-ellipsoid, brown to dark brown in 5% KOH, smooth, and germ pores are distinct. Basidia 13.0–16.4 × 6.7–8.0 μm, clavate, hyaline, and four or two-spored. Pileipellis is a two to three-layered irregular epithelium composed of subglobose cells, oval, (29.2)30.0–42.0(54.0) μm broad, and hyaline. Cheilocystidia is (17.8)20.9–27.7(31.5) × (11.6)12.3–14.9(16.0) μm, claviform to utriform, and rarely pyriform. Trama of gills is irregular. Pleurocystidia is absent.

Habit and habitat: solitary, in pairs, or scattered on the ground with rich humus in broad-leaved forests or broad-leaved shrubs. So far only found in China.

Other specimens examined: CHINA. Guizhou Province, Doupeng Mountain, 26°37′41″ N, 107°36′54″ E, 1057.8 m asl, 25 September 2021, *Yang Liu* and *Chen-Hao Li*, *ZRL20211843* (HMAS 258920).

**Figure 3:**
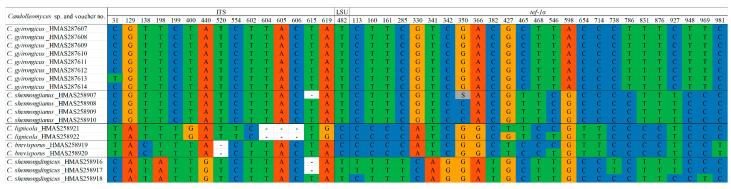
Nucleotide differences of *Candolleomyces brevisporus*, *C. shennongdingicus*, *C. gyirongicus*, *C. lignicola*, and *C. shennongjianus* across ITS, LSU, and *tef-1α*. The numbers at the top indicate the positions of the polymorphic sites in each fragment. The dashes indicate the lack of data for the respective positions.

Notes: *Candolleomyces brevisporus* is reminiscent of *C. subcacao* T. Bau & J.Q. Yan with its dirty white pileus and pale brown veil. Both were originally described in China, but *C. subcacao* differs from *C. brevisporus* by having larger basidiospores (6.8–8.0 × 3.9–4.9 μm), and longer basidia (17–22 × 6.1–7.3 μm) [11]. In the multigene tree (Figure 1), *Candolleomyces brevisporus* formed a monophyletic sister clade to *C. lignicola* with high support, but the former pileus is not hygrophanous, pale grey to greyish brown, and the two have different nrITS and *tef-1α* sequences (Figure 3).

**Figure 4:**
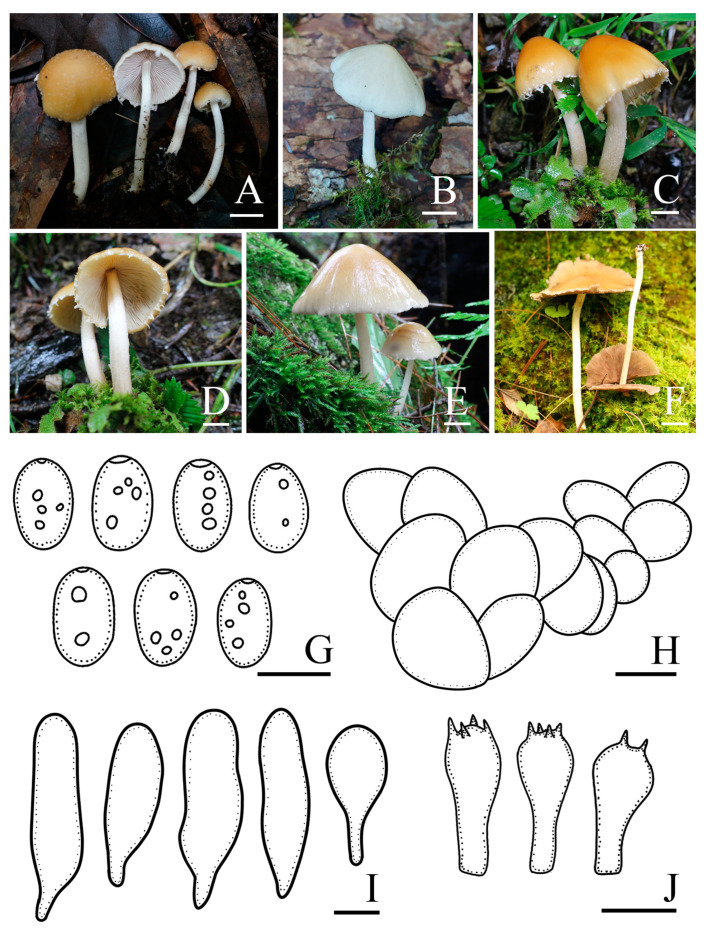
Basidiomata and microscopic features of *Candolleomyces gyirongicus*. (**A**–**F**) Basidiomata: (**A**) HMAS 287607; (**B**) HMAS 287610; (**C**,**D**) HMAS 287611; (**E**) HMAS 287612 (holotype); (**F**) HMAS 287614, (**G**) Basidiospores, (**H**) Pileipellis, (**I**) Cheilocystidia, and (**J**) Basidia. Scale bars: 10 mm (**A**–**F**); 5 μm (**G**); 20 μm (**H**); and 10 μm (**I**,**J**).

*Candolleomyces gyirongicus* R.L. Zhao, B. Cao & X.X. Han, sp. nov., Figure 4.

Fungal Names: FN 571921.

Holotype: CHINA. Xizang Autonomous Region, Shigatse Municipality, Gyirong County, Gyironggou, 28°24′ N, 85°18′ E, 2935 m asl, 1 August 2022, *Mao-Qiang He*, *Bin Cao*, *ZRL20220470* (holotype HMAS 287612). GenBank: PP734613 (nrITS), PP734624 (nrLSU), PP729326 (*tef-1α*).

Etymology: refers to Gyirong County, the locality of the type specimen.

Diagnosis: *Candolleomyces gyirongicus*, is distinguishable by its pileus, slightly hygrophanous. Basidiospores (5.5)6.1–6.9(8.0) × (3.2)3.8–4.3(4.7) μm, often with germ pores. Pileipellis is a one to two-layered irregular epithelium composed of subglobose cells. Cheilocystidia utriform, sometimes claviform.

Pileus is 15–56 mm diam, paraboloid to hemispherical when young, broadly conical, convex to broadly convex, becoming plano-convex to nearly plane when mature, slightly hygrophanous, with grey (5B1) veil elements at a young stage, white (5A1), orange-white (5A2) to golden brown (5D7), paler at the margin, and usually white (5A1) to orange-white (5A2). Veil is grey (5B1), fibrillose, and evanescent. Context is 0.2–0.5 mm broad at the centre, same color as pileus, and fragile. Lamellae is very close to moderately close, adnate to adnexed, orange-white (5A2) to pale orange (5A3) when immature, becoming greyish orange (5B5), and nougat (5D3) to greyish brown (6D3) when mature. Stipes are 30–100 × 3–6 mm, smooth, hollow, with white (5A1) fibrils at the base, and white (5A1) to yellowish white (3A2). Odour is not distinctive. Taste is indistinct.

Basidiospores are (5.5)6.1–6.9(8.0) × (3.2)3.8–4.3(4.7) μm, Q = 1.5–1.7, ellipsoid to oblong-ellipsoid, pale brown to dark brown in 5% KOH, smooth, often with germ pore. Basidia is 15.5–19.7 × 7.2–8.5 μm, clavate, hyaline, and four-spored. Pileipellis is a one to two-layered irregular epithelium composed of subglobose cells, (19.9)24.8–31.5(39.5) μm broad, and hyaline. Cheilocystidia is (25.7)37.7–53.1(61.1) × (7.1)9.1–12.7(15.1) μm, utriform, and sometimes claviform. Trama of gills is irregular. Pleurocystidia is absent.

Habit and habitat: solitary, scattered on soil, in bush, and broad-leaved or deciduous coniferous forest. So far only found in China.

Other specimens examined: CHINA. Yunnan Province, Jingdong County, Ailao Mountain, 24°52′ N, 101°03′ E, 2443 m asl, 4 July 2021, *Rui-Lin Zhao*, *Mao-Qiang He*, *Xin-Yu Zhu*, *Ming-Zhe Zhang*, *ZRL20210352* (HMAS 287607); Xizang Autonomous Region, Nyingchi Municipality, Zayü County, 28°36′ N, 98°05′ E, 4110 m asl, 21 July 2021, *Rui-Lin Zhao*, *Ming-Yu Zhu*, *Bin Cao*, *ZRL20210621* (HMAS 287608); Xizang Autonomous Region, Nyingchi Municipality, Mêdog County, Xironggou, 29°42′ N, 95°35′ E, 2800 m asl, 25 July 2021, *Bin Cao*, *Xin-Yu Zhu*, *Ming-Zhe Zhang*, *ZRL20210861* (HMAS 287609); Xizang Autonomous Region, Nyingchi Municipality, Mêdog County, Xironggou, 29°42′N, 95°35′ E, 2800 m asl, 25 July 2021, *Zhi-Lin Ling*, *Mao-Qiang He*, *ZRL20210966* (HMAS 287610); Xizang Autonomous Region, Shigatse Municipality, Dinggyê County, Chentang Town, Jiuyan hot spring, 27°55′ N, 87°21′ E, 3060 m asl, 29 July 2021, *Rui-Lin Zhao*, *Xin-Yu Zhu*, *ZRL20220325* (HMAS 287611); Xizang Autonomous Region, Shigatse Municipality, Gyirong County, Gyironggou, 28°26′ N, 85°15′ E, 3024 m asl, 1 August 2022, *Dorji Phurbu*, *Jia-Xin Li*, *ZRL20220628* (HMAS 287613); and Xizang Autonomous Region, Shigatse Municipality, Gyirong County, Gyironggou, 28°26′ N, 85°15′ E, 3024 m asl, 1 August 2022, *Dorji Phurbu*, *Jia-Xin Li*, *ZRL20220631* (HMAS 287614).

Notes: In the field, *Candolleomyces gyirongicus* is morphologically similar to *C. candolleanus*. However, *C. gyirongicus* can be distinguished from *C. candolleanus* by its smaller basidiospores, which measure (5.5)6.1–6.9(8.0) × (3.2)3.8–4.3(4.7) μm, and larger basidia (15.5–19.7 × 7.2–8.5 μm) [48,49]. In the multigene tree (Figure 1), *Candolleomyces gyirongicus* formed a monophyletic sister clade to *C. shennongjianus* with high support. However, *C. gyirongicus* has a narrower context, thinner stipe, as well as smaller basidiospores, slightly bigger basidia, and longer but narrower cheilocystidia. Additionally, there are some differences in the sequence of *tef-1α* (Figure 3). *Candolleomyces gyirongicus* is introduced as a new species based on morphology and phylogenetic analyses.

**Figure 5:**
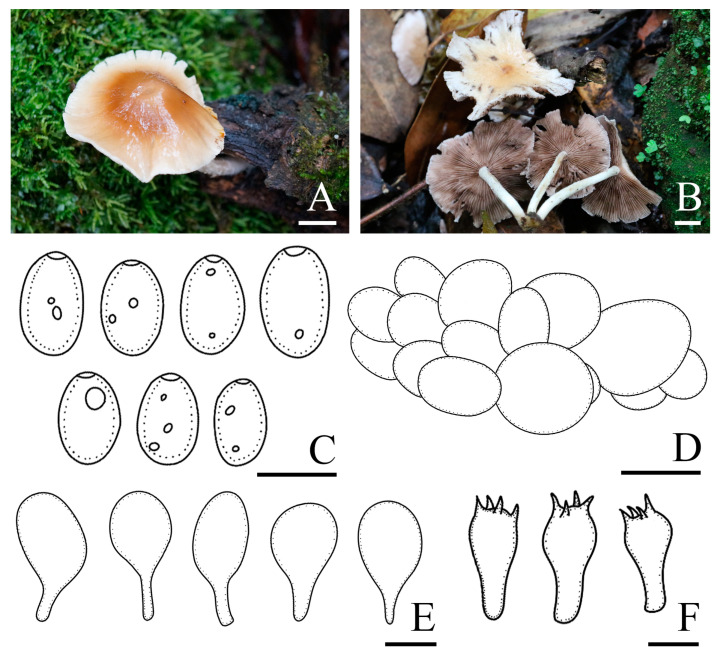
Basidiomata and microscopic features of *Candolleomyces lignicola*. (**A**,**B**) Basidiomata: (**A**) HMAS 258922; (**B**) HMAS 258921 (holotype), (**C**) Basidiospores, (**D**) Pileipellis, (**E**) Cheilocystidia, (**F**) Basidia. Scale bars: 10 mm (**A**,**B**); 5 μm (**C**); 20 μm (**D**); and 10 μm (**E**,**F**).

*Candolleomyces lignicola* R.L. Zhao, B. Cao & X.X. Han, sp. nov., Figure 5.

Fungal Names: FN571749.

Holotype: CHINA. Yunnan Province, Chuxiong, Zixi Mountain, 25°01′06″ N, 101°23′19″ E, 2235 m asl, 18 July 2021, *Rui-Lin Zhao*, *Bin Cao* and *Xin-Yu Zhu*, *ZRL20210496* (holotype HMAS 258921). GenBank: OR822169 (nrITS), OR822151 (nrLSU), OR819988 (*tef-1α*).

Etymology: ‘lignicola’ (Latin) refers to the habitat, this species grows mainly on rotting wood.

Diagnosis: *Candolleomyces lignicola* differs by its pileus, hygrophanous. Basidiospores (4.5)5.5–6.9(7.6) × (3.5)3.7–4.3(4.8) μm, a germ pore is absent or indistinct. Pileipellis is a two to three-layered irregular epithelium composed of irregular subglobose cells, irregular oval. Cheilocystidia claviform to somewhat broadly claviform or subsphaeropenduculate. Habitat on rotten wood.

Pileus has a 28–53 mm diam, flabellate, flattening with age, with or without obtuse umbo, hygrophanous, dark blonde (5D4) to yellowish brown (5D8) at the centre and golden blonde (5C4) to pale orange (5A3) toward the margin, becoming orange-white (5A2) as pileus dries, with split margins when mature. Veil is white (5A1), fibrillose, and evanescent. Context is thin and very fragile, the same colour as the pileus. Lamellae is close to moderately close, adnate to adnexed, nougat (5D3) to elay (5D5), and edge white (5A1) as basidiospores mature. Stipes are 23–51 × 3–9 mm, cylindrical, hollow, equal, white (5A1) to orange-white (5A2), and has a surface covered with yellowish white (3A2) fibrillose. Odour is not distinctive.

Basidiospores are (4.5)5.5–6.9(7.6) × (3.5)3.7–4.3(4.8) μm, Q = 1.4–1.7, ellipsoid to oblong-ellipsoid, pale brown to brown in 5% KOH, smooth, a germ pore is absent or indistinct. Basidia is 12.1–15.8 × 6.1–7.9 μm, clavate, hyaline, and 4 or 2-spored. Pileipellis is a two to three-layered irregular epithelium composed of irregular subglobose cells, irregular oval, (19.0)24.0–35.8(43.6) μm broad, and hyaline. Cheilocystidia is (18.4)22.1–32.1(40.6) × (7.5)10.7–15.3(18.9) μm, claviform to somewhat broadly claviform or subsphaeropenduculate, and rarely with deposits. Trama of gills is irregular. Pleurocystidia is absent.

Habit and habitat: solitary, in pairs, or scattered on rotten wood in broad-leaved forests or broad-leaved shrubs.

Other specimens examined: CHINA. Yunnan Province, Nangunhe Nature Reserve, 23°22′06″ N, 99°21′22″ E, 1633 m asl, 3 July 2021, *Rui-Lin Zhao*, *Mao-Qiang He*, and *Ming-Zhe Zhang*, *ZRL20210404* (HMAS 258922).

Notes: *Candolleomyces lignicola* can easily be mistaken for *C. yanshanensis* in the field due to their similar macroscopic characteristics. However, *C. yanshanensis* differs from *C. lignicola* due to its slightly larger basidiospores (5.8–8.2 × 3.3–5.4 μm) and longer basidia (17–31 × 5.8–7.5 μm) [12]. *Candolleomyces lignicola* distinguishes itself from the sister species *C. brevisporus* by its broader pileus, wider lamellae, and longer and thicker stipes. Additionally, there are differences in their nrITS and *tef-1α* sequences (Figure 3). Notably, *Candolleomyces lignicola* was collected on wood rather than soil.

**Figure 6:**
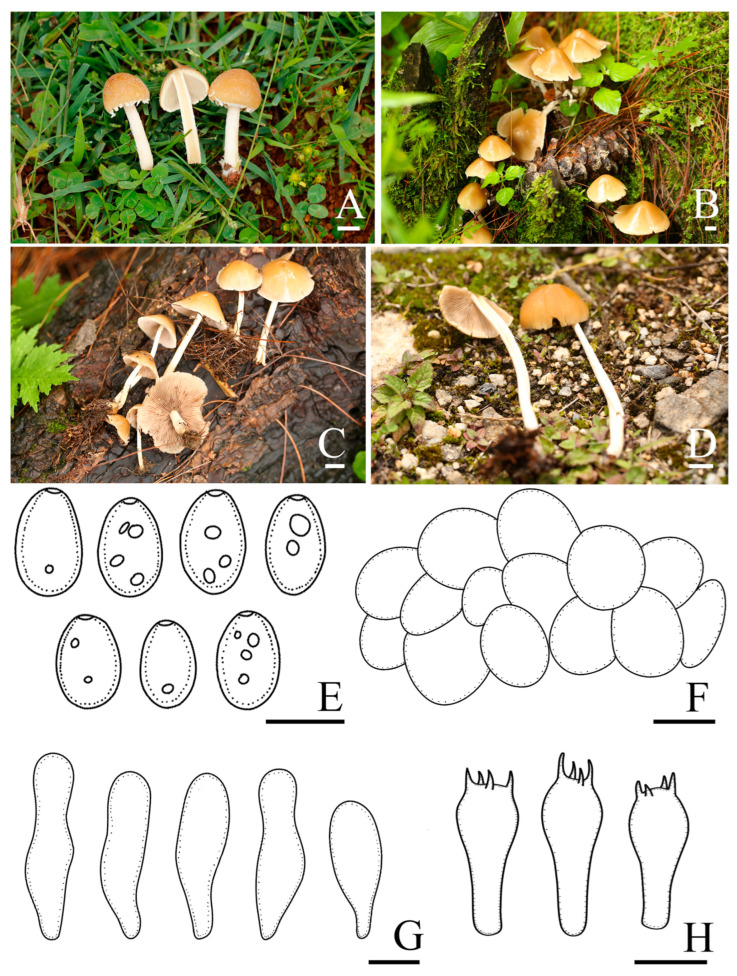
Basidiomata and microscopic features of *Candolleomyces luridus*. (**A**–**D**) Basidiomata: (**A**) HMAS 258913, (**B**) HMAS 258913 (holotype), (**C**) HMAS 258914, (**D**) HMAS 258915, (**E**) Basidiospores, (**F**) Pileipellis, (**G**) Cheilocystidia, and (**H**) Basidia. Scale bars: 10 mm (**A**–**D**); 5 μm (**E**); 20 μm (**F**); and 10 μm (**G**,**H**).

*Candolleomyces luridus* R.L. Zhao, B. Cao & X.X. Han, sp. nov., Figure 6.

Fungal Names: FN571751.

Holotype: CHINA. Xizang Autonomous Region, Shigatse Municipality, Gyirong County, Gyironggou, 28°14′24″ N, 85°10′48″ E, 2935 m asl, 1 August 2022, *Dorji Phurbu* and *Jia-Xin Li*, *ZRL20220606* (holotype HMAS 258913). GenBank: OR822161 (nrITS), OR822143 (nrLSU), OR819980 (*tef-1α*). 

Etymology: ‘luridus’ (Latin) refers to the yellowish brown colours of the pileus.

Diagnosis: *Candolleomyces luridus* is distinguished by its pileus, hygrophanous. Basidiospores are (5.3)6.1–7.1(8.3) × (3.4)3.9–4.6(5.2) μm, germ pores are distinct. Pileipellis a one to two-layered irregular epithelium composed of irregular subglobose cells, that is an irregular oval. Cheilocystidia is narrowly utriform to utriform, sometimes subclaviform. 

Pileus is 21–50 mm diam, broadly conical when young and convex when mature, with or without obtuse umbo, hygrophanous, golden yellow (5B7) to yellowish brown (5D8), with orange-white (5A2) veil elements at a young stage, striate up to halfway from the margin or indistinct, sometimes cleft or lobed. Veil is white (5A1), fibrillose, and falls off easily. Context is thin and very fragile, the same colour as the pileus. Lamellae is adnate to adnexed, pale orange (5A3) to elay (5D5), and edge is orange-white (5A2) to white (5A1) as basidiospores mature. Stipes are 39–72 × 3–8 mm, cylindrical, hollow, equal, orange-white (5A2) to pale orange (5A3), with a surface covered with slight white fibrils, and is evanescent. Odour is not distinctive.

Basidiospores are (5.3)6.1–7.1(8.3) × (3.4)3.9–4.6(5.2) μm, Q = 1.4–1.7, ellipsoid to oblong-ellipsoid, pale brown to brown in 5% KOH, smooth, and germ pores are distinct. Basidia is 16.7–19.5 × 7.3–8.6 μm, clavate, hyaline, and four or two-spored. Pileipellis is a one to two-layered irregular epithelium composed of irregular subglobose cells, is an irregular oval, (14.3)22.0–36.0(42.9) μm broad, and hyaline. Cheilocystidia is (20.6)26.0–41.4(53.8) × (7.6)9.0–11.8(14.4) μm, narrowly utriform to utriform, sometimes subclaviform, and rarely with deposits. Trama of gills is irregular. Pleurocystidia is absent.

Habit and habitat: in pairs, scattered or clustered on humus-rich ground or decaying wood in broad-leaved or deciduous coniferous forests.

Other specimens examined: CHINA. Xizang Autonomous Region, Shigatse Municipality, Gyirong County, Gyironggou, 28°14′24″ N, 85°10′48″ E, 2935 m asl, 1 August 2022, *Dorji Phurbu* and *Jia-Xin Li*, *ZRL20220625* (HMAS 258914) and *ZRL20220627* (HMAS 258915); Sichuan Province, Liangshan Yi Autonomous Prefecture, Yanyuan County, Xiamosuogou, 27°39′31″ N, 101°16′6″ E, 1953 m asl, 8 August 2019, *Rui-Lin Zhao*, *Bin Cao*, and *Zhi-Lin Ling*, *ZRL20190449* (HMAS 258912); Sichuan Province, Ganzi Tibetan Autonomous Prefecture, Xiangcheng County, Fozhuxia Nature Reserve, 29°3′53″ N, 99°56′16″ E, 3090 m asl, 21 August 2020, *Rui-Lin Zhao* and *Xi-Xi Han*, *ZRL20201771* (HMAS 258911); Beijing City, Miyun District, Taishitun Town, Bailongtan, 40°29’32″ N, 117°4’0″ E, 302 m asl, 28 August 2023, *Bin Cao, Ming-Yu Zhu* and *Bei Han*, *ZRL20230723* (HMAS 287929); and Beijing City, Pinggu District, Laoquankou Village, 40°29’32″ N, 117°4’0″ E, 229 m asl, 15 August 2023, *Jia-Xin Li*, *Wen-Qiang Yang* and *Ze-Zhi Wang*, *ZRL20233312* (HMAS 287930).

Notes: *Candolleomyces luridus* is easily confused with *C. candolleanus* and *C. gyirongicus* in the field due to its similar macroscopic characteristics, but *C. candolleanus* differs from *C. luridus* in having larger basidiospores (7–8 × 4.5–5.5 μm) and smaller basidia (14–17 × 6–7 μm) [48,49], while *C. gyirongicus* has a longer cheilocystidia. The nrITS and *tef-1α* sequences of *C. luridus* are distinct from other members of *Candolleomyces* (Figure 3). Therefore, we introduce *C. luridus* as a new species based on morphology and phylogenetic analyses (Figure 1, Figure 2 and Figure 6).

**Figure 7:**
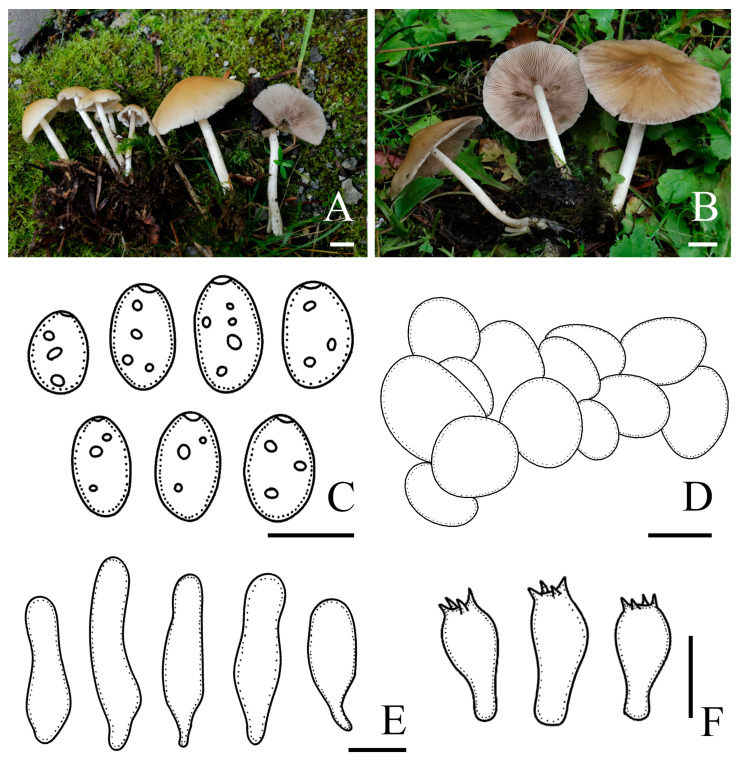
Basidiomata and microscopic features of *Candolleomyces shennongdingicus*. (**A**,**B**) Basidiomata: (**A**) HMAS 258918 (holotype); (**B**) HMAS 258916, (**C**) Basidiospores, (**D**) Pileipellis, (**E**) Cheilocystidia, (**F**) Basidia. Scale bars: 10 mm (**A**,**B**); 5 μm (**C**); 20 μm (**D**); 10 μm (**E**,**F**).

*Candolleomyces shennongdingicus* R.L. Zhao, B. Cao & X.X. Han, sp. nov., Figure 7.

Fungal Names: FN571750.

Holotype: CHINA. Hubei Province, Shennongjia National Park, Shennongding, Jinhou Ridge, 31°16′48″ N, 110°10′47.9″ E, 2498 m asl, 25 August 2022, *Rui-Lin Zhao* and *Mao-Qiang He*, *ZRL20220855* (holotype HMAS 258918). GenBank: OR822166 (nrITS), OR822148 (nrLSU), OR819985 (*tef-1α*).

Etymology: ‘shennongdingicus’ refers to the location Shennongding where the holotype was collected.

Diagnosis: *Candolleomyces shennongdingicus* is recognised by the pileus, hygrophanous. Basidiospores are (5.3)6.2–7.4(8.5) × (3.4)3.8–4.3(4.7) μm, and germ pores are distinct but small. Pileipellis is a one to two-layered irregular epithelium composed of subglobose cells. Cheilocystidia is narrowly utriform, seldom cylindrical to claviform.

Pileus has a 25–53 mm diam, parabolic when young and convex when mature, with or without obtuse umbo, hygrophanous, is darker in the centre, golden yellow (5B7) to golden brown (5D7) at the centre and golden blonde (5C4) to nougat (5D3) toward the margin, with striate up to halfway from the margin or indistinct. Veil is white (5A1), fibrillose, and gradually disappearing in later stages. Context is grey (5C1), thin, and very fragile. Lamellae is adnate, moderately close, orange-grey (5B2) to grey (5D1), and the edge becomes white as basidiospores mature. Stipes are 34–53 × 3–5 mm, cylindrical, hollow, equal, and white (5A1) to orange-white(5A2), with the same color flocculent fibres. Odour is not distinctive.

Basidiospores are (5.3)6.2–7.4(8.5) × (3.4)3.8–4.3(4.7) μm, Q = 1.5–1.8, ellipsoid to oblong, brown to dark brown in 5% KOH, smooth, the germ pore is distinct but small. Basidia 15.8–21.2 × 6.4–7.9 μm, short clavate, hyaline, 4 or 2-spored. Pileipellis is a one to two-layered irregular epithelium composed of subglobose cells, (14.9)19.8–29.5(37.4) μm broad, hyaline. Cheilocystidia (23.8)29.8–40.5(49.1) × (8.2)9.5–13.3(15.3) μm, narrowly utriform, seldom cylindrical to claviform, thin-walled, rarely with deposits. Trama of gills is irregular. Pleurocystidia is absent.

Habit and habitat: solitary, scattered or clustered on the ground with rich humus in broad-leaved or deciduous coniferous forests.

Other specimens examined: CHINA. Xizang Autonomous Region, Shigatse Municipality, Dinggyê County, Chentang Town, Xiaerba Village, 27°31′12″ N, 87°15′0″ E, 2600 m asl, 30 July 2022, *Dorji Phurbu*, *ZRL20220339* (HMAS 258917); Xizang Autonomous Region, Shigatse Municipality, Dinggyê County, Chentang Town, Jiuyan hot spring, 27°33′0″ N, 87°12′36″ E, 3060 m asl, 29 July 2022, *Mao-Qiang He*, *Bin Cao*, *Jia-Xin Li*, *ZRL20220411* (HMAS 258916).

*Notes:* In the field, *Candolleomyces shennongdingicus* can be easily confused with *C. shennongjianus* at first glance, as both species have parabolic pileus when young and convex when mature, and yellowish brown pileus. Additionally, both species exhibit white stipes with pale yellowish brown bases. However, *C. shennongdingicus* can be distinguished by its slightly smaller basidiospores, longer basidia, as well as fusiform, seldom cylindrical to clavate, and smaller cheilocystidia. Moreover, phylogenetic analysis reveals that *C. shennongdingicus* is distinct from *C. shennongjianus* (Figure 3). Based on morphology and phylogenetic analyses, *Candolleomyces shennongdingicus* is introduced as a new species (Figure 1 and Figure 7).

**Figure 8:**
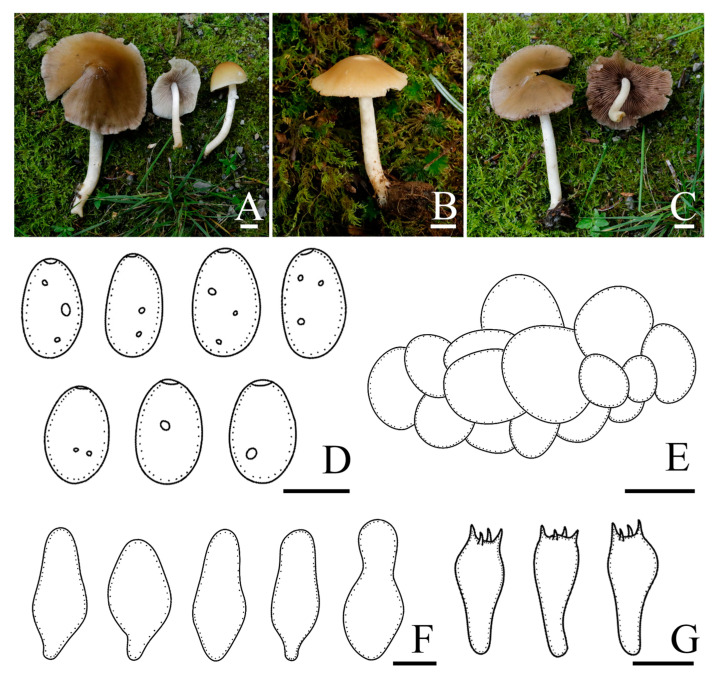
Basidiomata and microscopic features of *Candolleomyces shennongjianus*. (**A**–**C**) Basidiomata: (**A**) HMAS 258909 (holotype); (**B**) HMAS 258907; (**C**) HMAS 258910, (**D**) Basidiospores, (**E**) Pileipellis, (**F**) Cheilocystidia, and (**G**) Basidia. Scale bars: 10 mm (**A**–**C**); 5 μm (**D**); 20 μm (**E**); and 10 μm (**F**,**G**).

*Candolleomyces shennongjianus* R.L. Zhao, B. Cao & X.X. Han, sp. nov., Figure 8.

Fungal Names: FN571748.

Holotype: CHINA. Hubei Province, Shennongjia National Park, Shennongding, Jinhou Ridge, 31°16′48″ N, 110°10′47.9″ E, 2498 m asl, 25 August 2022, *Rui-Lin Zhao*, *Mao-Qiang He*, *ZRL20220858* (holotype HMAS 258909). GenBank: OR822157 (nrITS), OR822139 (nrLSU), OR819976 (*tef-1α*).

Etymology: *shennongjianus* refers to the location Shennongjia National Park, where the type specimen was collected.

Diagnosis: *Candolleomyces shennongjianus* is distinguishable by its pileus, hygrophanous. Basidiospores are (5.9)6.7–8.4(9.4) × (3.9)4.2–4.9(5.3) μm, the germ pore is distinct but small. Pileipellis is a two to three-layered irregular epithelium composed of irregular subglobose cells, and is a irregular oval. Cheilocystidia is utriform, subclaviform, and sometimes pyriform.

Pileus is 23–63 mm diam, paraboloid when young, obtusely conical, convex, or plano-convex when mature, with or without obtuse umbo, and sometimes cleft or lobed; surface is glabrous, dull, hygrophanous, oak brown (5D6) to bronze (5E5), darker in the centre, and striate up to halfway from the margin or indistinct. Veil is white (5A1), dispersed, fibrillose, and falls off easily. Context is 0.5–1.0 mm broad at the centre, the same colour as pileus. Lamellae is moderately close, adnate to slightly adnexed, grey (5C1), brownish orange (5C3) to hair brown (5E4), and the edge becomes white as basidiospores mature. Stipes are 45–70 × 4–9 mm, sometimes with occasional white flocculation, hollow, white (5A1) to pale orange (5A3), and sometimes dark blond (5D4) at the base. Odour is indistinct.

Basidiospores are (5.9)6.7–8.4(9.4) × (3.9)4.2–4.9(5.3) μm, Q = 1.5–1.8, ellipsoid to oblong, brown (#b06500) to dark brown (#4f484c) in 5% KOH, abundant, smooth, germ pores are distinct but small. Basidia is 13.3–18.3 × 6.6–8.7 μm, clavate, hyaline, and four-spored. Pileipellis is a two to three-layered irregular epithelium composed of irregular subglobose cells, is an irregular oval, (15.7)19.2–28.8(38.0) μm broad, and hyaline. Cheilocystidia is (27.5)35.0–45.3(51.3) × (8.2)11.2–14.7(16.9) μm, utriform, subclaviform, sometimes pyriform, rarely with deposits, and thin-walled. Trama of gills is irregular. Pleurocystidia is absent.

Habit and habitat: Solitary, in pairs, or scattered on the ground with rich humus in broad-leaved or deciduous coniferous forests.

Other specimens examined: CHINA. Hubei Province, Shennongjia National Park, Shennongding, Jinhou Ridge, 31°16′48″ N, 110°10′47.9″ E, 2498 m asl, 25 August 2022, *Rui-Lin Zhao* and *Mao-Qiang He*, *ZRL20220857* (HMAS 258910); and Hubei Province, Shennongjia National Park, Shennongding, Guanyin Cave, 31°17′24″ N, 110°10′12″ E, 2283 m asl, 2 September 2022, *Rui-Lin Zhao*, *Bin Cao*, *Xi-Xi Han* and *Xin-Yu Zhu*, *ZRL20221427* (HMAS 258907) and *ZRL20221467* (HMAS 258908).

Notes: *Candolleomyces shennongjianus* is morphologically similar to *C. asiaticus*. However, *C. asiaticus* can be distinguished by its broader basidiospores (7.2–7.6 × 4.5–6 vs. 6.7–8.4 × 4.2–4.9 μm), larger basidia (19.3–22.5 × 9.4–10.5 vs. 13.3–18.3 × 6.6–8.7 μm), and shorter cheilocystidia (21–38 × 9.6–16 vs. 35.0–45.3 × 11.2–14.7 μm) [14]. In addition, there are differences in their nrITS and *tef-1α* sequences (Figure 3). Based on morphological characteristics and phylogenetic analyses, *C. shennongjianus* is introduced as a new species (Figure 1, Figure 3 and Figure 8).

**Figure 9:**
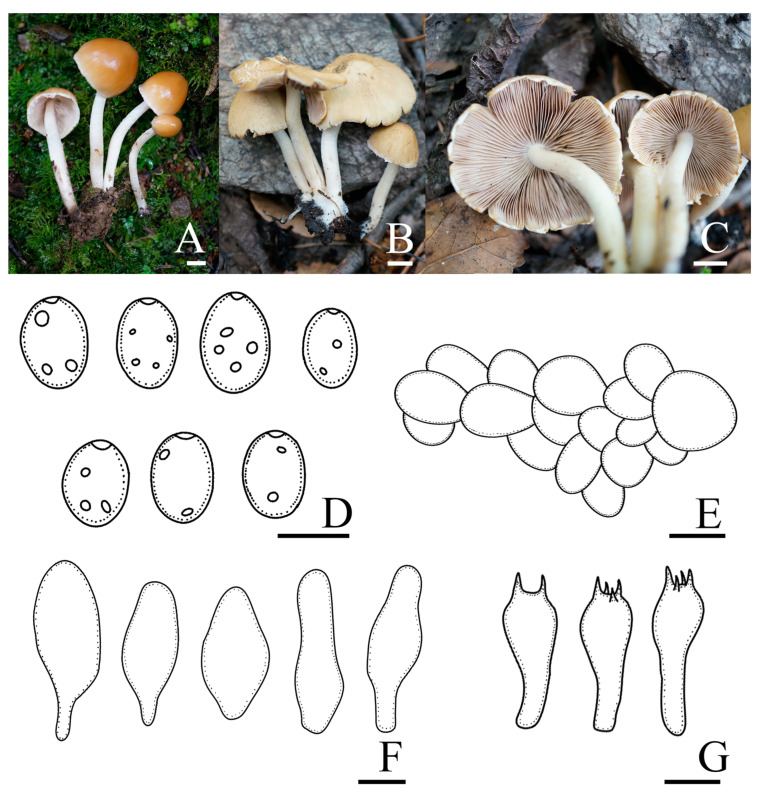
Basidiomata and microscopic features of *Candolleomyces sichuanicus*. (**A**–**C**) Basidiomata: (**A**) HMAS 287615; (**B**,**C**) HMAS 287616 (holotype), (**D**) Basidiospores, (**E**) Pileipellis, (**F**) Cheilocystidia, and (**G**) Basidia. Scale bars: 10 mm (**A**–**C**); 5 μm (**D**); 20 μm (**E**); and 10 μm (**F**,**G**).

*Candolleomyces sichuanicus* R.L. Zhao, B. Cao & X.X. Han, sp. nov., Figure 9.

Fungal Names: FN 571922.

Holotype: CHINA. Sichuan Province, Ganzi Tibetan Autonomous Prefecture, Derong County, Xiayong Nature Reserve, 28°22′ N, 99°21′ E, 3399 m asl, 22 August 2020, *Bin Cao*, *Jia-Xin Li*, *ZRL20201861* (holotype HMAS 287616). GenBank: PP734617 (nrITS), PP734628 (nrLSU), PP729330 (*tef-1α*).

Etymology: refers to Sichuan Province, the locality of the type specimen.

Diagnosis: *Candolleomyces sichuanicus* differs from other species by its pileus, not hygrophanous. Basidiospores is (5.6)6.4–7.9(8.6) × (3.4)4.0–4.8(5.1) μm, sometimes germ pores are absent. Pileipellis a two to three-layered irregular epithelium composed of irregular subglobose cells, and is an irregular oval. Cheilocystidia is utriform, rarely subclaviform.

Pileus is 8–42 mm diam, paraboloid to hemispherical when young and convex to plano-convex when mature, sometimes cleft or lobed, moist, smooth, not hygrophanous, not striate to rimos, and golden blonde (5C4) to yellowish brown (5D8). Veil is white (5A1), fibrillose, and evanescent. Context is thin and very fragile, and the same colour as the pileus. Lamellae is adnexed, grey (5B1) to nougat (5D3), and the edge becomes white (5A1) as spores mature. Stipes are 21–68(74) × 3–7 mm, hollow, and white (5A1) to grey (5B1). Odour is not distinctive. Taste is indistinct.

Basidiospores are (5.6)6.4–7.9(8.6) × (3.4)4.0–4.8(5.1) μm, Q = 1.5–1.8, ellipsoid to oblong, pale brown to brown in water, abundant, smooth, and sometimes germ pores are absent. Basidia is 17.4–22.4 × 7.9–8.9 μm, clavate, hyaline, and four-spored. Pileipellis is a two to three-layered irregular epithelium composed of irregular subglobose cells, is an irregular oval, (12.2)17.9–26.3(33.2) μm broad, and hyaline. Cheilocystidia is (27.7)35.0–44.6(55.1) × (9.9)11.7–15.1(17.8) μm, utriform, rarely subclaviform. Trama of gills is irregular. Pleurocystidia is absent.

Habit and habitat: Scattered or clustered on the ground with rich humus in broad-leaved or deciduous coniferous forests. So far only found in China in July/August.

Other specimens examined: CHINA. Sichuan Province, Ganzi Tibetan Autonomous Prefecture, Yajiang County, Gexigou National Nature Reserve, 30°3′ N, 100°56′ E, 2953 m asl, 15 August 2020, *Rui-Lin Zhao*, *Ming-Zhe Zhang*, *Mei-Qi Wang*, *ZRL20200271* (HMAS 287615).

Notes: *Candolleomyces sichuanicus* is morphologically similar to *C. cladii-marisci* and *C. gyirongicus*. *Candolleomyces cladii-marisci* differs from *C. sichuanicus* by having larger basidiospores (7–9.5 × 4–5.5 vs. 6.4–7.9 × 4.0–4.8 μm), smaller basidia (8.5–20.5 × 6–9 vs. 17.4–22.4 × 7.9–8.9 μm), and narrower cheilocystidia (21.5–54 × 6–11 vs. 35.0–44.6 × 11.7–15.1 μm) [13]. In contrast, *Candolleomyces gyirongicus* can be distinguished by its longer stipe, smaller basidiospores, shorter basidia, and longer but narrower cheilocystidia. Phylogenetic analysis and morphological characteristics supported the proposal of this new species (Figure 1, Figure 3 and Figure 9).


**Key to Candolleomyces species distributed in Chinese**
1a Spores very pale, nearly hyaline in 5% KOH21b Spores pale yellowish brown, greyish brown or darker82a Spores mostly larger than 8.0 μm32b Spores less than 8.0 μm43a Spores larger than 8.5 μm and mostly wider than 5.0 μm
*C. luteopallidus*
3b Not as above
*C. sulcatotuberculosus*
4a Spores less than 7.0 μm54b Not as above75a Basidiomata slender, spores nearly hyaline in water
*C. subminutisporus*
5b Basidiomata stout, spores orange-white to pale orange in water66a Pileus 5–20 mm, brown to golden brown
*C. subcandolleanus*
6b Pileus 5–25 mm, incanus to nude
*C. incanus*
7a Basidiomata stout, spores up to 5.5 μm wide
*C. singeri*
7b Basidiomata slender, spores up to 4.5 μm wide
*C. subsinger*
8a Spores larger than 10.0 μm
*C. typhae*
8b Not as above99a Spores without a germ pore, sequestrate basidoma and marine habits109b Spores with a germ pore1110a Brownish basidoma
*C. brunneovagabundus*
10b Whitish basidoma
*C. albovagabundus*
11a Sometimes germ pore absent1211b Germ pore distinct1512a Pileipellis isa one to two-layered irregular epithelium
*C. gyirongicus*
12b Pileipellis is a two to three-layered irregular epithelium1313a Spores up to 5.0 μm wide
*C. yanshanensis*
13b Not as above1414a Cheilocystidia claviform to somewhat broadly claviform or subsphaeropenduculate
*C. lignicola*
14b Cheilocystidia utriform, rarely subclaviform
*C. sichuanicus*
15a Spores up to 5.0 μm wide, larger than 8.0 μm
*C. leucotephrus*
15b Not as above1616a Spores less than 4.0 μm wide, pileus 6–22 mm
*C. albipes*
16b Spores 3.7–4.9 μm wide, pileus 10–100 mm1717a Basidiomata slender, pileus yellowish grey to grey, brown, becoming white as dries1817b Basidiomata stout, orange-white, golden yellow to yellowish brown1918a Spores less than 6.8 μm
*C. brevisporus*
18b Not as above
*C. subcacao*
19a Spores less than 7.5 μm2019b Spores up to 7.5 μm, germ pore distinct but small2120a Spores 6.2–7.4 × 3.8–4.3 μm, germ pore distinct but small
*C. shennongdingicus*
20b Spores 6.1–7.1 × 3.9–4.6 μm, germ pore distinct
*C. luridus*
21a Pileus 23–63 mm, spores 6.7–8.4 × 4.2–4.9 μm
*C. shennongjianus*
21b Pileus 10–100 mm, spores 6.1–9.0 × 3.7–4.5 μm
*C. candolleanus*


## 4. Discussion

At present, 15 species in *Candolleomyces* were reported from China viz. *C. albipes* [50], *C. albovagabundus* [19], *C. brunneovagabundus* [19], *C. candolleanus* [21], *C. incanus* [12], *C. leucotephrus* [21], *C. luteopallidus* [21], *C. singer* [21], *C. subcacao* [11], *C. subcandolleanus* [12], *C. subminutisporus* [11], *C. subsingeri* [21], *C. sulcatotuberculosus* [11], *C. typhae* [21], and *C. yanshanensis* [12]. Yan (2018) reported the distribution of *C. leucotephrus*, *C. singer*, and *C. subsingeri* in China based on morphological characteristics and ITS sequences [21]. Subsequently, in 2021, Bau and Yan supplemented these specimens with LSU, *tef-1α*, and *β-Tub* sequences [11]. Additionally, they identified the sample with the voucher of HFJAU1515 as *C. sulcatotuberculosus* and provided its ITS, *tef-1α*, and *β-Tub* sequences but did not describe its morphology [11]. Furthermore, *Psathyrella typhae* var. *bispora* was reported as a new variety in China in 2018 [21], and it became synonymised with *Candolleomyces typhae* [21]. However, only its SSU sequence is available in NCBI, lacking the complete sequence data needed to verify its presence in China through phylogenetic analysis. On the other hand, *C. albipes* has only morphological descriptions in China, with no associated molecular data, leaving its actual presence in the region uncertain and requiring further investigation [50]. Furthermore, although *C. singeri* was previously reported in Hubei, this paper marks the first record of the species in the Shennongjia National Park. In the recorded 43 *Candolleomyces* species, 36 species have nrITS sequences, 27 species have nrLSU sequences, and 16 species have *tef-1α* sequences. However, only 14 species have nrITS, nrLSU, and *tef-1α* sequences. All five new species revealed in this study provided sequences of nrITS, nrLSU, and *tef-1α*.

Significant progress for *Candolleomyces* was made in the study, but there are still some challenges. Existing classifications primarily rely on a combination of morphological characters and molecular data [18,20]. However, due to the variability of morphological traits and the limited availability of gene sequence data, the identification and classification of certain species remain problematic [9]. In recent years, the development of molecular biology techniques, such as *tef-1α* and *β-tub* gene sequence analyses, greatly facilitated systematic taxonomic studies of the genus [11,12,19]. The ongoing discovery of well-defined boundaries in new taxa, as demonstrated by this study, enhances our understanding of species within this genus. In addition, the edible and medicinal values, as well as the toxicity, of only a few species, such as *C. candolleanus*, *C. tuberculatus*, and *C. yanshanensis*, were clarified, while those of most other species remain unknown. Meanwhile, the ecological functions and distribution ranges of the majority of species are still ambiguous. The diversity of the genus *Candolleomyces* continues to increase with the discovery of new species, necessitating more comprehensive field investigations, as well as morphological and molecular studies to refine the taxonomic system [12]. Future research should focus on integrating morphological, molecular, and ecological methods to further elucidate the phylogenetic relationships and species diversity within the genus.

## 5. Supplementary Note

In the paper “Zhi-Lin Yuan, Fu-Cheng Lin, Chu-Long Zhang, Christian P. Kubicek, A new species of *Harpophora* (Magnaporthaceae) recovered from healthy wild rice (*Oryza granulata*) roots, representing a novel member of a beneficial dark septate endophyte [51], the authors propose corrections for the invalid publication name, which does not conform to Nom. inval., Art. 40.7 (Melbourne). Two strain numbers are provided, where the holotypes lyophilised culture no. R5-6-1 was deposited. These were corrected as follows: China General Microbiological Culture Collection Center (CGMCC 2737) was designated as the holotype, and Centraalbureau voor Schimmelcultures (CBS 125863) was designated as the paratype.

*Harpophora oryzae* Z.L. Yuan, C.L. Zhang & F.C. Lin

Holotype: CGMCC 2737

Paratype: CBS 125863

## Figures and Tables

**Table 1:** Sequences used in the phylogenetic analysis in this study. Missing sequences are indicated by “–”.

Taxon	Voucher	Country	nrITS	nrLSU	*tef-1α*	Reference
*Candolleomyces aberdarensis*	GLMF116094	Kenya	MH880928	–	–	[39]
*C. aberdarensis*	IHIB618	Kenya	MK421517	MK421517	–	[10]
*C. albipes*	DED8340	Sao Tome	KX017209	–	–	[40]
*C. albosquamosus*	TBGT18600 Type	India	OQ676550	–	–	[15]
*C. albosquamosus*	TBGT18895	India	OQ676549	–	–	[15]
*C. albovagabundus*	HTBM1139 Paratype	China	OR711038	OR711054	–	[19]
*C. albovagabundus*	HKAS129660 Type	China	OR711041	OR711057	OR727285	[19]
*C. asiaticus*	LAH36975	Pakistan	OK392606	OQ802843	–	[14]
*C. asiaticus*	LAH36809 Type	Pakistan	NR182405	NG229114	–	[14]
*C. badhyzensis*	TAA79478 Type	Turkmenistan	KC992883	KC992883	–	[30]
*C. badiophyllus*	SZMC-NL-2347	-	FN430699	FM876268	FM897252	[41]
*C. bivelatus*	110114MFBPZHO777	China	MW554021	–	–	Non-referenced
*C. bivelatus*	MCVE29117	Italy	MF325962	–	MF521811	[42]
***C. brevisporus***	**HMAS 258919 Type**	**China**	**OR822167**	**OR822149**	**OR819986**	**This study**
***C. brevisporus***	**HMAS 258920**	**China**	**OR822168**	**OR822150**	**OR819987**	**This study**
*C. brunneopileatus*	TBGT18553	India	OQ878483	OR244398	–	[20]
*C. brunneopileatus*	TBGT18698 Type	India	OQ878348	OR244401	–	[20]
*C. brunneovagabundus*	HKAS129659 Type	China	OR711031	OR711047	OR791600	[19]
*C. brunneovagabundus*	HTBM1136 Paratype	China	OR711036	OR711052	–	[19]
*C. cacao*	FP1R4	USA	KU847452	–	–	Non-referenced
*C. cacao*	DED 8339 Type	Sao Tome	NR148106	–	–	[40]
*C. campanulatus*	LAH35719 Type	Pakistan	OQ308881	OQ802837	–	[18]
*C. campanulatus*	LAH37657	Pakistan	OQ308882	OQ802838	–	[18]
*C. candolleanus*	LAS73030 Neotype	Sweden	KM030175	KM030175	–	[30]
*C. candolleanus*	LÖ38-00	Sweden	DQ389720	DQ389720	–	[5]
*C. cladii-marisci*	CLUF302 Type	Italy	MK080112	–	–	[43]
*C. cladii-marisci*	SDBR-CMUNK0507	Thailand	MZ145228	MZ145244	–	[13]
*C. efflorescens*	Pegler2133(K)	Sri Lanka	KC992941	–	–	[30]
*C. eurysporus*	GLMF126263 Type	Viet Nam	MT651560	MT651560	–	[10]
***C. gyirongicus***	**HMAS 287607**	**China**	**PP734608**	**PP734619**	**PP729321**	**This study**
***C. gyirongicus***	**HMAS 287608**	**China**	**PP734609**	**PP734620**	**PP729322**	**This study**
***C. gyirongicus***	**HMAS 287609**	**China**	**PP734610**	**PP734621**	**PP729323**	**This study**
***C. gyirongicus***	**HMAS 287610**	**China**	**PP734611**	**PP734622**	**PP729324**	**This study**
***C. gyirongicus***	**HMAS 287611**	**China**	**PP734612**	**PP734623**	**PP729325**	**This study**
***C. gyirongicus***	**HMAS 287612 Type**	**China**	**PP734613**	**PP734624**	**PP729326**	**This study**
***C. gyirongicus***	**HMAS 287613**	**China**	**PP734614**	**PP734625**	**PP729327**	**This study**
***C. gyirongicus***	**HMAS 287614**	**China**	**PP734615**	**PP734626**	**PP729328**	**This study**
*C. halophilus*	MICH AH-14321	Spain	MG825900	–	–	[44]
*C. incanus*	BJTC S173	China	ON042760	ON042767	ON098509	[12]
*C. incanus*	BJTC Z777 Type	China	ON042759	ON042766	ON098508	[12]
*C. leucotephrus*	LÖ138-01 (UPS)	Sweden	KC992885	KC992885	KJ732775	[30]
*C. leucotephrus*	MCVE28705	Spain	MF325979	–	MF521791	[42]
***C. lignicola***	**HMAS 258921 Type**	**China**	**OR822169**	**OR822151**	**OR819988**	**This study**
***C. lignicola***	**HMAS 258922**	**China**	**OR822170**	**OR822152**	**OR819989**	**This study**
***C. luridus***	**HMAS 258911**	**China**	**OR822159**	**OR822141**	**OR819978**	**This study**
***C. luridus***	**HMAS 258912**	**China**	**OR822160**	**OR822142**	**OR819979**	**This study**
***C. luridus***	**HMAS 258913 Type**	**China**	**OR822161**	**OR822143**	**OR819980**	**This study**
***C. luridus***	**HMAS 258914**	**China**	**OR822162**	**OR822144**	**OR819981**	**This study**
***C. luridus***	**HMAS 258915**	**China**	**OR822163**	**OR822145**	**OR819982**	**This study**
*C. luteopallidus*	HMJAU5148	China	MG734736	MW301084	MW314073	[45]
*C. luteopallidus*	Sharp20863 Type	USA	KC992884	KC992884	–	[30]
*C. niveofloccosus*	TBGT18412 Type	India	OQ878345	OR244387	–	[20]
*C. niveofloccosus*	TBGT18513	India	OQ878251	OR244394	–	[20]
*C. ruhunensis*	HKAS123158 Type	Sri Lanka	ON685315	–	–	[17]
*C. secotioides*	AH31746 Type	Mexico	KR003281	KR003282	KR003283	[7]
***C. shennongdingicus***	**HMAS 258916**	**China**	**OR822164**	**OR822146**	**OR819983**	**This study**
***C. shennongdingicus***	**HMAS 258917**	**China**	**OR822165**	**OR822147**	**OR819984**	**This study**
***C. shennongdingicus***	**HMAS 258918 Type**	**China**	**OR822166**	**OR822148**	**OR819985**	**This study**
***C. shennongjianus***	**HMAS 258907**	**China**	**OR822155**	**OR822137**	**OR819974**	**This study**
***C. shennongjianus***	**HMAS 258908**	**China**	**OR822156**	**OR822138**	**OR819975**	**This study**
***C. shennongjianus***	**HMAS 258909 Type**	**China**	**OR822157**	**OR822139**	**OR819976**	**This study**
***C. shennongjianus***	**HMAS 258910**	**China**	**OR822158**	**OR822140**	**OR819977**	**This study**
***C. sichuanicus***	**HMAS 287615**	**China**	**PP734616**	**PP734627**	**PP729329**	**This study**
***C. sichuanicus***	**HMAS 287616 Type**	**China**	**PP734617**	**PP734628**	**PP729330**	**This study**
***C. sichuanicus***	**HMAS 287617**	**China**	**PP734618**	**PP734629**	**PP729331**	**This study**
*C. sindhudeltae*	LAH37632 Type	Pakistan	OQ247908	OQ247912	–	[16]
*C. sindhudeltae*	LAH37633	Pakistan	OQ247909	OQ247913	–	[16]
*C. singeri*	HMJUA37867	China	MG734718	MW301088	MW314077	[45]
*C. singeri*	HMJAU37877	China	MW301073	MW301091	MW314080	[11]
***C. singeri***	**HMAS 258923**	**China**	**OR822171**	**OR822153**	**OR819990**	**This study**
***C. singeri***	**HMAS 258924**	**China**	**OR822172**	**OR822154**	**OR819991**	**This study**
*C. subcacao*	HMJAU37807 Type	China	MW301064	MW301092	MW314081	[11]
*C. subcacao*	HMJAU37808	China	MW301065	MW301093	MW314082	[11]
*C. subcandolleanus*	BJTC Z239 Type	China	ON042755	ON042762	ON098505	[12]
*C. subcandolleanus*	BJTC Z232	China	ON042756	ON042763	–	[12]
*C. subminutisporus*	HMJAU37801 Type	China	MW301066	MW301094	MW314083	[11]
*C. subminutisporus*	HMJAU37916	China	MW301067	MW301095	MW314084	[11]
*C. subsingeri*	HMIAU37913	China	MG734725	MW301098	MW314086	[45]
*C. subsingeri*	HMJAU37811 Type	China	MG734715	MW301097	MW314085	[45]
*C. sulcatotuberculosus*	LÖ55-12	Germany	KJ138422	KJ138422	–	[46]
*C. sulcatotuberculosus*	HFJAU1515	China	MW375696	–	MW382965	[11]
*C. sultanii*	LAH35714 Type	Pakistan	OQ308835	OQ801565	–	[18]
*C. sultanii*	LAH37658	Pakistan	OQ308836	OQ801566	–	[18]
*C. thailandensis*	SDBR-CMUNK0442	Thailand	MZ145232	–	–	[13]
*C. thailandensis*	SDBR-CMUNK0443 Type	Thailand	MZ146874	–	–	[13]
*C. trinitatensis*	TL9035	Ecuador	KC992882	KC992882	–	[30]
*C. tuberculatus*	ADK4162	Sweden	KC992886	KC992886	–	[30]
*C. typhae*	LÖ21-04	Sweden	DQ389721	DQ389721	KJ732776	[5]
*C. yanshanensis*	BJTC Z783	China	ON042757	ON042764	ON098506	[12]
*C. yanshanensis*	BJTC Z110 Type	China	ON042758	ON042765	ON098507	[12]
*Hausknechtia floriformis*	WU22832 Type	Vanuatu	ON745613	ON745616	ON746007	[47]
*H. floriformis*	WU22833	Vanuatu	ON745619	ON745615	ON746009	[47]
*H. leucosticta*	HFJAU1486 Epitype	China	OL435561	OL435565	OL439896	[47]
*H. leucosticta*	HFJAU1526	China	OL435563	OL435566	OL439897	[47]

The sequences generated in this study are marked in bold.

## Data Availability

All sequence data are available in NCBI GenBank following the accession numbers in the manuscript.
